# Modeling the within-host dynamics of *Plasmodium vivax* hypnozoite activation: An analysis of the SPf66 vaccine trial

**DOI:** 10.1073/pnas.2401024121

**Published:** 2024-12-10

**Authors:** Somya Mehra, François Nosten, Christine Luxemburger, Nicholas J. White, James A. Watson

**Affiliations:** ^a^Mahidol Oxford Tropical Medicine Research Unit, Faculty of Tropical Medicine, Mahidol University, Bangkok 10400, Thailand; ^b^School of Mathematics and Statistics, The University of Melbourne, Parkville, VIC 3010, Australia; ^c^Shoklo Malaria Research Unit, Mae Sot, Tak, 63110, Thailand; ^d^Centre for Tropical Medicine and Global Health, Nuffield Department of Medicine, University of Oxford, Oxford OX3 7LF, United Kingdom; ^e^Infectious Diseases Data Observatory, Big Data Institute, Oxford OX3 7LF, United Kingdom

**Keywords:** hypnozoite, within-host, mathematical model, vivax malaria, exponential clock

## Abstract

*Plasmodium vivax* parasites can lie dormant in the liver as hypnozoites, activating weeks to months after sporozoite inoculation to cause relapsing malarial illness. It is not known what biological processes govern hypnozoite activation. We use longitudinal data from the most detailed cohort study ever conducted in an area where both *Plasmodium falciparum* and *P. vivax* were endemic to fit a simple within-host mathematical model of *P. vivax* hypnozoite activation. 1,344 children living on the Thailand–Myanmar border were followed daily for 21 mo. There were 2,504 vivax and 1,164 falciparum malaria symptomatic episodes recorded over 1988 person-years. The model assumes that hypnozoites activate independently at a constant rate (“exponential clock model”). When this model was embedded in a stochastic framework for repeated infectious mosquito bites, with seasonality inferred from the incidence of clinical falciparum malaria episodes, it explained the observed temporal patterns of multiple (up to 13) recurrent vivax malaria episodes. Under this model, we estimate the mean dormancy period for a single hypnozoite to be 6 mo (i.e., a half-life of 4 mo). We use the calibrated within-host model to characterize population-level overdispersion in the risk of relapse, and assess the potential utility of a serological test for radical cure in low transmission settings. We show that mefloquine treatment of falciparum malaria eliminates early vivax relapses; and that there are substantially more *P. vivax* recurrences than expected under the model following artesunate monotherapy treatment for falciparum malaria. These results suggest that hypnozoites can be activated by symptomatic malarial illness.

## Significance

*Plasmodium vivax* is the main cause of human malaria outside Africa. Relapse, caused by the activation of dormant liver hypnozoites, is a major cause of symptomatic illness and transmission. Little is known about the mechanisms of hypnozoite activation. We calibrate a simple mathematical model of relapse, which assumes random constant-rate activation of individual hypnozoites, to longitudinal data from the most detailed cohort study ever conducted in an area co-endemic for both vivax and falciparum malaria. We show that this model captures most of the inter- and intra-individual variability in recurrence patterns, yielding falsifiable insights into parasite biology. Controlling for confounding by previous exposure, we provide evidence supporting the hypothesis that hypnozoite activation can be triggered by symptomatic falciparum malaria.

*Plasmodium vivax* and *Plasmodium ovale* malaria parasites are characterized by their ability to lie dormant in liver cells for variable periods, with periodic activation leading to relapsing bloodstream infections. This has allowed *P. vivax* to extend into temperate regions and persist in people during periods which are unfavorable for the mosquito vectors (1). Prevention of relapse requires treatment with 8-aminoquinoline antimalarials (“radical cure”), which cause hemolytic toxicity in patients with glucose-6-phosphate dehydrogenase (G6PD) deficiency (2), making elimination of this important parasite difficult (3).

The biology of relapse is not well understood. How hypnozoites activate in the liver remains largely unknown (4). A major challenge is the difficulty in developing appropriate in vitro platforms to study the fundamental biology of *P. vivax* (5, 6). A key question is how much variation in hypnozoite activation is driven by intrinsic vs. extrinsic stimuli. There are at least two subspecies of *P. vivax*: The prevalent short latency “tropical” vivax which relapses at short intervals—although the frequency and intervals vary geographically—and a “temperate” long latency vivax which has an interval of approximately 8 to 9 mo between either inoculation and the primary illness, or between the primary illness and the first relapse (7, 8).

An intrinsic biological clock mechanism is likely to explain long latency in temperate strains of *P. vivax*. Several external triggers of activation have been proposed for both short and long latency strains. These range from seasonal changes (9) and the bites of potential malaria vectors (10) to the febrile inflammatory response (11) and hemolysis (12). One hypothesis is that each febrile malaria episode itself (regardless of the species) can trigger subsequent hypnozoite activation (3). This hypothesis was based on the observation that the risk of vivax malaria after acute falciparum malaria in both adults and children enrolled in therapeutic studies in South East Asia was nearly as high as the risk after acute vivax malaria, and the intervals between acute illness and relapse were similar (13, 14): i.e., in therapeutic studies a falciparum malaria episode was highly predictive of a vivax relapse. As mosquito dual infections (i.e. mosquitoes infected by both malaria species) are relatively rare in low transmission settings (15), this suggested that falciparum malaria could trigger hypnozoite activation (3). It has been theorized that an external sensing mechanism may be coupled with spontaneous activation at a low baseline rate (3, 4).

Mathematical models of vivax relapse in combination with human data cannot prove or disprove a given biological theory. Further, mathematical and biological parsimony do not have the same heuristic values: Biological parsimony can be considered from the viewpoint of likelihood of selection or evolution but need not have a simple mathematical formulation. The mathematically simplest “base” model of vivax relapse was formulated by Michael White and colleagues: a constant activation rate per hypnozoite, with independent and identical dynamics for each hypnozoite and no external triggers (16). This results in an exponentially distributed time to vivax malaria relapse, with a rate conditional on the hypnozoite burden. We refer to this as the “exponential clock” model. If this simple model could predict patterns of relapse robustly within and across individuals, this would provide some evidence in favor of the corresponding biological theory, with the caveat that biological theories and mathematical models do not have a one-to-one equivalence. In addition, this simple model would have practical utility if it could explain the majority of the inter- and intra-individual variability in relapse risk in endemic areas. For example, it would help understand where to target drug administration campaigns for radical cure.

In this work, we derive analytical likelihoods for the exponential clock model allowing us to infer the model parameters from longitudinal data on recurrent infections. We fit the model to data from a very detailed prospective trial of a *P. falciparum* vaccine which proved ineffective. This was conducted in an area of low seasonal malaria transmission co-endemic for both *P. falciparum* and *P. vivax* (17). In this trial, 1,344 children aged 2 to 15 y of age living in a refugee camp located on the North-Western border of Thailand were seen each day for 21 mo. This is the most detailed cohort study of vivax malaria ever conducted. We show that in this epidemiological setting the exponential clock model can capture patterns of multiple recurrent infections over time across childhood age groups, with up to 13 symptomatic vivax episodes recorded per child. We found higher than expected rates of vivax after *P. falciparum* monoinfections treated with artesunate monotherapy, which is compatible with an external triggering mechanism. We also found that unlike chloroquine (18), mefloquine treatment provides sufficient drug exposure for one month to eliminate some emerging relapses, a previously unappreciated public health benefit. This post-treatment prophylactic effect may have masked any triggering effects as most falciparum episodes were treated with artesunate-mefloquine. We use the fitted model to quantify overdispersion in relapse risk and characterize the potential utility of a serological test to guide radical cure.

## Results

### Modeling Hypnozoite Activation As an Exponential Clock.

The exponential clock model is the simplest mathematical model describing hypnozoite activation. Under this model, each hypnozoite activates independently with an exponentially distributed time to activation with constant rate η. To capture the observed variation in recurrence patterns, we add stochasticity in i) the timing of infectious mosquito bites (non-homogeneous Poisson process); ii) the inoculated sporozoite batch sizes (assumed geometric distribution); and iii) sporozoite “destiny” (hypnozoite vs. immediately developing form: binomial distribution, giving rise to correlation in the burden of primary infection and relapse associated with each bite). Finally, we model the acquisition of anti-disease immunity using age as a proxy for previous malaria exposure. Anti-disease immunity is defined as the probability that a bloodstream malaria infection results in symptomatic illness and is modeled as a flexible decreasing deterministic function of age. An overview of model parameters is given in Table 1.

**Table 1.** Summary of within-host model parameters

Parameter	Interpretation
η	Rate of activation of each hypnozoite (the inverse is the expected duration of hypnozoite carriage)
ν	Mean size of successful sporozoite inoculum (i.e. number of sporozoites that undergo immediate development or form a viable hypnozoite that is destined to activate)
$p_{\;\text{rel}}$	Probability that a successful sporozoite becomes a hypnozoite that will subsequently activate ($1-p_{\;\text{rel}}$ undergo immediate development)
λ(t)	Force of inoculation (i.e. infective anopheline vector bite rate per human) at time t
ρ	Proportional reduction in the probability of a symptomatic bloodstream *P. vivax* infection for each hypnozoite activation and immediate sporozoite development event between the ages of 2 and 15 y
γ	Shape coefficient for the age-dependent anti-disease masking probability

We show that the model likelihood is analytically tractable when formulated as an open network of infinite server queues (*SI Appendix*, Appendices B.1 and B.2 and ref. 19). Although very simple, this within-host model gives rise to complex temporal patterns of infection. There are three key structural features.

First, the exponential clock mechanism results in a linear relationship between the relapse rate in an individual and the size of their hypnozoite reservoir. Both the expected time to next relapse, and the variation in the time to next relapse, scale inversely with the hypnozoite burden (19). This results in short inter-relapse intervals initially following an infectious bite, with subsequent lengthening of the inter-relapse intervals as the hypnozoite reservoir is depleted. This pattern is consistent with observations from artificial infection studies (20, 21). Under this model relapses occurring in quick succession can be explained by either a high activation rate or a large hypnozoite burden.

Second, the number of hypnozoites carried by individuals in low transmission areas (loosely defined as one *P. vivax* infectious bite per year, which approximates most vivax malaria endemic areas outside of Oceania) is highly variable (overdispersed). This results from variation in the number and timing of infectious bites (infrequent inoculation), and variation in the number of viable sporozoites inoculated per bite (19). We expect zero inflation in the distribution of hypnozoite burdens in young children, persisting for a time scale determined by the force of inoculation (in particular, the rate at which infants are exposed to infectious mosquito bites) (19). As the child grows under a constant force of inoculation, repeated mosquito sporozoite inoculation, and thus hypnozoite accumulation, is eventually balanced out by hypnozoite activation, resulting in a steady-state distribution for the hypnozoite burden (19).

Third, there is a nonlinear relationship between age and the number of symptomatic relapses, arising as a consequence of the competing time scales of hypnozoite accrual and acquisition of anti-disease immunity which attenuates or prevents the symptoms of malaria during relapse. Hypnozoite accrual is determined by the expected duration of hypnozoite carriage. The acquisition of anti-disease immunity is determined by the force of inoculation, and access to treatment, but is modeled indirectly as a deterministic function of age. Age-dependent differences in mosquito inoculation rates will also result in age-dependent differences in hypnozoite burdens (22, 23).

### The SPf66 Vaccine Trial.

**Fig. 1.**
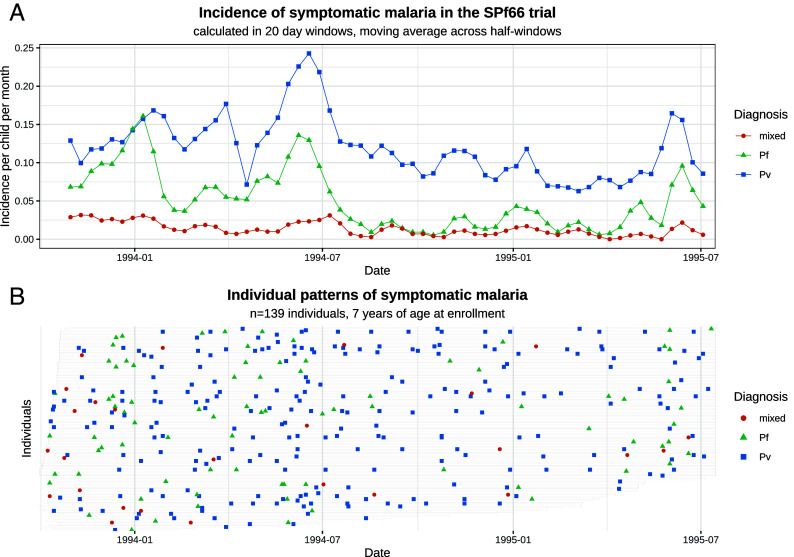
Symptomatic falciparum and vivax malaria in the SPf66 trial. Panel (*A*) shows the aggregate monthly incidence of symptomatic malaria (blue: *P. vivax*; green: *P. falciparum*; orange: mixed infections). Mixed infections are double-counted as vivax and falciparum episodes. Panel (*B*) shows individual data from all participants aged 7 y at enrollment (the gray bar shows the period of active detection; documented absences from the camp are not indicated).

The SPf66 vaccine trial (1993 to 1995) was the largest and most detailed cohort study of vivax malaria ever conducted. This was a randomized controlled double-blind trial assessing a synthetic protein vaccine candidate for falciparum malaria (17). The vaccine was shown to have no clinical efficacy. In the study 1,344 children between 2 and 15 y of age, living in a camp for displaced persons situated on the Thailand–Myanmar border, were followed daily for symptoms over 21 mo. Most of the children were of the Karen ethnic group. This represents an unusual epidemiological setting, with a stable population, relatively constrained movement, and malaria transmission occurring primarily within the camp (<1 km^2^) (17). There were twice-yearly peaks in incidence during the rainy season (May to June) and cool season (November to December; Fig. 1*A*) (24). Around April 1994 artesunate-mefloquine combination therapy became the first line treatment for falciparum malaria throughout the camp, resulting in a marked decline in falciparum malaria transmission (25) (Fig. 1*A*).

In study participants, malaria was diagnosed by microscopy and promptly treated under supervision. Typically, vivax monoinfections were treated with chloroquine monotherapy (25mg base/kg over 3 d), while uncomplicated falciparum monoinfections and mixed infections were treated with artesunate (4 mg/kg for 3 d) and mefloquine (25 mg/kg on day 2 of treatment) combination therapy. Artesunate monotherapy for five days was given to some children with hyperparasitemic falciparum infections (see *SI Appendix*, Appendix A.1 for details). Severe malaria cases were treated with artemether (intramuscular administration of 3.2 mg/kg on day 1, then 1.6 mg/kg per day), followed by oral artesunate-mefloquine combination therapy. Inter-consultation intervals show that chloroquine and mefloquine conferred little post-treatment prophylactic protection against falciparum malaria (*SI Appendix*, Fig. A.1), consistent with contemporaneous evidence of widespread multidrug-resistant *P. falciparum* (26). Radical cure with primaquine was not standard of care at the time, and only a few children with very frequently relapsing vivax infections were given primaquine. Access to antimalarial drugs was tightly regulated (25), and self-treatment was very unlikely.

This study provides detailed data on the timing and frequency of symptomatic *P. vivax* and *P. falciparum* infections in a low-transmission co-endemic setting. After screening for clear treatment failures (n=13 *P. vivax* monoinfections, n=13 *P. falciparum* monoinfections and n=1 mixed infection; *SI Appendix*, Appendix A.1), a total of 2,504 symptomatic *P. vivax* and 1,164 symptomatic *P. falciparum* infections were observed over 1988 person-years follow-up, of which 293 (9%) were mixed infections (*SI Appendix*, Table A.3). Up to 13 symptomatic vivax and 7 symptomatic falciparum episodes were recorded for each child (Fig. 1*B*). Consistent with previous reports (3), age structure in the incidence of symptomatic malaria (*SI Appendix*, Appendix A.4) suggests that sustained exposure would have been sufficient to confer partial anti-disease immunity against *P. vivax*, but not *P. falciparum*, in the age range studied (2 to 15 y of age).

### Calibration to Longitudinal Recurrence Data from the SPf66 Trial.

We fitted our within-host model to all symptomatic vivax episodes observed in the SPf66 trial. *P. falciparum* infections were used to estimate seasonality. Since the trial was conducted predominantly in school-aged children in the unusual epidemiological setting of a large refugee camp, we made the simplifying assumption of homogeneity in transmission intensity. The model accounted for post-treatment prophylaxis, including an extended period of protection following mefloquine treatment during which the bloodstream infection is eliminated; and a “bunching” effect following chloroquine treatment (27), whereby bloodstream infections are variably delayed resulting in aggregation of times to patent relapse (*SI Appendix*, Appendix A.1). All assumptions relevant to model fitting are outlined in Box 1, with detailed model fitting results provided in *SI Appendix*, Appendix C.1.

Box 1. Exponential clock model assumptions

There are four structural assumptions in the model:

1. Each hypnozoite has an independent exponentially distributed time to activation, with no external triggers.
2. The number of inoculated sporozoites is geometrically distributed.^∗^
3. The ratio of immediately developing sporozoites to hypnozoites is 6:4 [informed by recent in vivo experiments on the Chesson strain of *P. vivax* (28)].^∗^
4. The probability of clinical symptoms for each bloodstream infection (primary or relapse) decays monotonically as a function of age (i.e. age is considered a proxy for prior exposure and thus immunity), ignoring within-batch genetic relatedness, and with all cases assumed to be symptomatic in 2 y olds.

There are four assumptions in the observational model which are context dependent (dependent on drug pharmacokinetics, drug resistance patterns, and transmission):

1. Each treatment is associated with a fixed period of subsequent prophylactic protection, during which all emerging bloodstream infections are eliminated. For *P. vivax*, this is 15 d following chloroquine, 35 d following mefloquine, and 15 d following artesunate or quinine monotherapy.
2. Chloroquine provides an additional fixed period delaying (but not eliminating) *P. vivax* multiplication in the blood.
3. There is no age-stratification or heterogeneity in the force of inoculation for *P. vivax* between birth and 15 y of age.^∗^
4. Seasonal fluctuations in the incidence of symptomatic falciparum malaria are a proxy for seasonal fluctuations in the force of inoculation for *P. vivax*.

^∗^The impact of model misspecification was assessed for these assumptions

#### Duration of hypnozoite carriage.

**Fig. 2.**
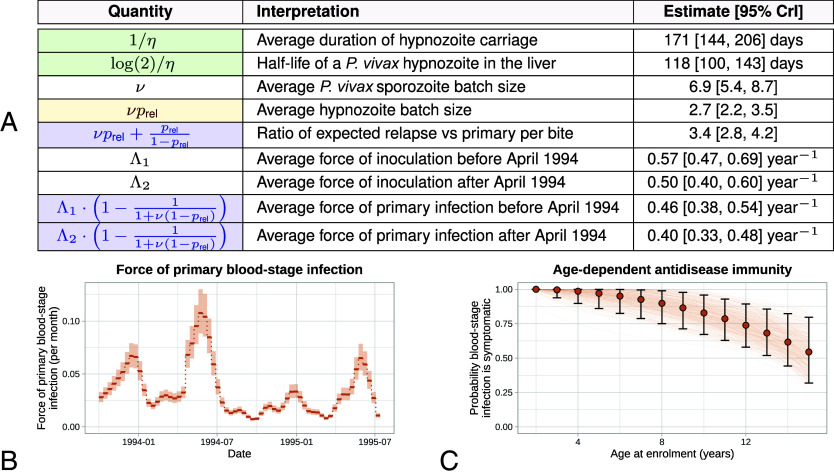
Summary of posterior model estimates. Panel (*A*) posterior median estimates [95% credible intervals (CrI)] for quantities of epidemiological interest. Blue: estimates robust to misspecification in the sporozoite batch distribution; yellow: estimates robust to misspecification of the hypnozoite fating probability; green: estimates robust to both forms of misspecification. Panel (*B*) force of primary blood-stage infection, with seasonality inferred from the incidence of symptomatic falciparum malaria over 10 d windows (shaded regions show 95% CrI). Scaling factors for the ratio of vivax to falciparum inoculation rates are estimated separately before and after April 1994 (day 200 of the study) when there was a camp-wide shift to artesunate-mefloquine treatment of falciparum malaria. Panel (*C*) age-dependent probability of symptomatic bloodstream vivax malaria infection, relative to a 2 y old. Error bars indicate 95% CrI for each age group; continuous curves are plotted for 200 parameter combinations (ρ,γ) sampled from the posterior distribution.

Patterns of recurrent symptomatic vivax malaria in the SPf66 trial were strongly informative for the hypnozoite activation rate η. Under the model, we estimated a hypnozoite activation rate of 1/171 d^−1^ (95% credible interval [CrI] 1/206 to 1/144 d^−1^), amounting to a half-life of 118 d (95% CrI 100 to 143 d) for a population of hypnozoites (Fig. 2*A*). Detailed simulation studies showed that these estimates were robust to misspecification of the sporozoite batch size (i.e. under- or over-dispersion of the size of the sporozoite inocula relative to the geometric distribution, *SI Appendix*, Appendix D.1), in addition to misspecification of the ratio of immediately developing forms to hypnozoites (*SI Appendix*, Appendix D.3). The estimated duration of hypnozoite carriage for the SPf66 trial (171 d) is similar to previous estimates for South East Asian strains (∼190 d), obtained by calibrating the exponential clock model with geometrically distributed hypnozoite loads to data for the time to first relapse (16).

#### Sporozoite inocula.

The mean number of successful sporozoites inoculated per infective bite was estimated to be 6.9 (95% CrI 5.4 to 8.7) when assuming a ratio of immediately activating forms to hypnozoites of 6:4 (Fig. 2*A*). This estimate is a lower bound for the true mean sporozoite batch size, since hypnozoite death is unidentifiable based on longitudinal recurrence data (*SI Appendix*, Appendix B.1). The corresponding estimate for the average number of hypnozoites per bite was 2.7 (95% CrI 2.2 to 3.5). However, this estimate was largely insensitive to the assumed ratio of immediately developing forms to hypnozoites (*SI Appendix*, Appendix D.3).

#### Relative burden of relapse and primary infection.

The ratio of relapsing to primary *P. vivax* infections within the cohort was estimated as 3.4 (95% CrI 2.8 to 4.2) (Fig. 2*A*), i.e. ∼75% of observed symptomatic *P. vivax* infections were relapses. This is lower than previous estimates of the relative relapse burden (∼95%) in patients seeking treatment for vivax malaria in the same transmission setting after treatment for their initial enrollment episode (29, 30). One possible explanation is that these previous estimates only included recurrent episodes in their calculation and ignored the enrollment episode. This biases the estimate to a higher proportion of relapses. In a low transmission setting, the enrollment episode is less likely to be a relapse. Estimates of the ratio of relapsing to primary infection and the force of primary bloodstream infection (Fig. 2*B*) were robust to misspecification of the sporozoite batch size distribution (*SI Appendix*, Appendix D.1). Thus, based on inter-recurrence intervals, we can classify individual episodes probabilistically as relapses or reinfections (*SI Appendix*, Appendix E.4).

#### Impact of the assumption of homogeneity on estimates.

Our simulation studies indicate that unmeasured heterogeneity in the force of inoculation can lead to the systematic overestimation of sporozoite batch sizes ν; and underestimation of the force of inoculation and the hypnozoite activation rate η (*SI Appendix*, Appendix D.2). However, the extent of bias is heavily dependent on the degree of transmission heterogeneity. Under a negative binomial model, we estimate that 43% of symptomatic falciparum episodes (95% CrI: 40% to 47%) occurred in the top 20% of children (*SI Appendix*, Appendix A.5). We interpret this as an upper bound for transmission heterogeneity: While the incidence of symptomatic falciparum malaria is a correlate of mosquito inoculation, temporal variation in follow-up periods means that the apparent heterogeneity in incidence was likely driven in part by seasonal effects and the sustained decline in transmission intensity in the second half of the study period (Fig. 1*A*). Under this degree of heterogeneity, our simulation studies suggest that the hypnozoite activation rate η is unlikely to be underestimated by more than 30% (average duration of carriage 120 vs. 170 d); while the unbiased sporozoite batch size is unlikely to be less than 5 sporozoites (the model estimates approximately 7). We thus argue that the assumption of homogeneity in the force of inoculation does not fundamentally alter our findings.

### The Relationship Between Age and Symptomatic Vivax Malaria.

**Fig. 3.**
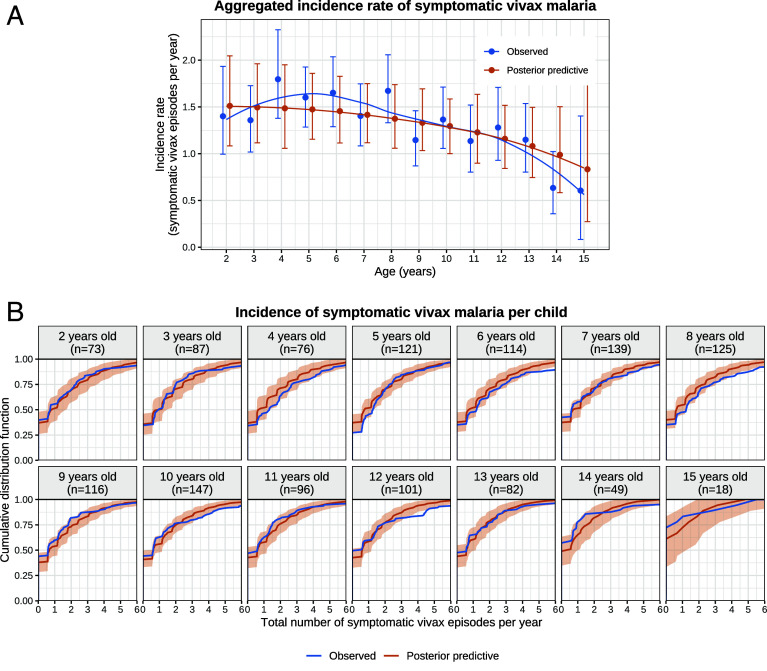
Characterizing the vivax malaria model fit by age group. Panel (*A*) shows the observed vs. posterior predictive incidence rate of symptomatic vivax malaria, stratified by age at enrollment; error bars show 95% bootstrap CIs for observed data, and 95% posterior predictive intervals. Panel (*B*) shows the cumulative distribution functions of the incidence of symptomatic vivax malaria per child. Shaded areas: 95% credible intervals. Simulation of data under the posterior predictive distribution is described in *SI Appendix*, Appendix C.2.1.

If the average duration of dormancy for each hypnozoite is 6 mo (half-life of 4 mo), then the hypnozoite reservoir would reach steady state after approximately 2 y of life under a constant force of inoculation (19). In the absence of age-stratification in the force of inoculation, this implies that the number of hypnozoites was distributed identically across all age groups in the SPf66 trial: While there would have been variation in the hypnozoite burden across individuals, systematic differences across age groups would not be expected.^*^ Slowly eliminated antimalarial treatments affect the intervals between symptomatic relapses because they reduce asexual stage multiplication. As age-related differences in pharmacokinetics are considered negligible, the model fit implies that all age structure in the incidence of symptomatic vivax was determined by anti-disease immunity alone. This should result in a monotonic decline in the incidence of symptomatic vivax across age groups (Fig. 2*C*). However, the data showed a non-monotonic relationship between age and symptomatic vivax malaria (Fig. 3*A*). Peak incidence occurred around the age of four ($P=1\times 10^{-4}$ using an asymptotic bootstrap for the Mack-Wolfe test for umbrella alternatives with unknown peak, *SI Appendix*, Appendix A.4). There was a clear discrepancy between the model fit and observed data for the age-stratified *P. vivax* incidence (Fig. 3*B*). This discrepancy may be explained by age-dependent differences in the frequency of mosquito inoculation in the first years of life. It is unlikely that there were substantial differences in the force of inoculation for children >2 y as there is no evidence for age structure in the incidence of symptomatic falciparum malaria (P=0.8 for the Kruskal–Wallis test, *SI Appendix*, Appendix A.4), a more direct correlate of mosquito inoculation. However, infants under 2 y may have had lower exposure to infectious mosquito bites (likely resulting from their lower mobility or smaller size compared with older children), leading to fewer hypnozoites accrued by age 2 (31, 32). We therefore refitted the model with the force of inoculation for infants under 2 y scaled down by a series of fixed factors. Estimates for the hypnozoite activation rate, the mean sporozoite batch size, the force of inoculation, and age-dependent anti-disease masking characteristic were unchanged; but the description of the non-monotonic age structure in the incidence of symptomatic vivax malaria was improved (*SI Appendix*, Appendix D.4).

### Vivax Malaria following Falciparum Malaria.

Several studies have described higher than expected rates of vivax relapse following symptomatic falciparum malaria (13, 14). We assessed whether the exponential clock model without external triggering could satisfactorily explain the proportion of symptomatic *P. vivax* malaria episodes observed after symptomatic *P. falciparum* monoinfections in the SPf66 trial. We compared model-predicted vs. observed vivax recurrence rates following *P. falciparum* monoinfections, accounting for the documented history of vivax recurrence. Model-predicted rates of vivax recurrence are dependent on correct modeling of seasonality which was a limitation in our model fit.^†^ Overall, the observed rates of vivax recurrence were largely compatible with model predictions at fixed time points (i.e. irrespective of *P. falciparum* infection) throughout the trial (*SI Appendix*, Fig. C.4).

**Fig. 4.**
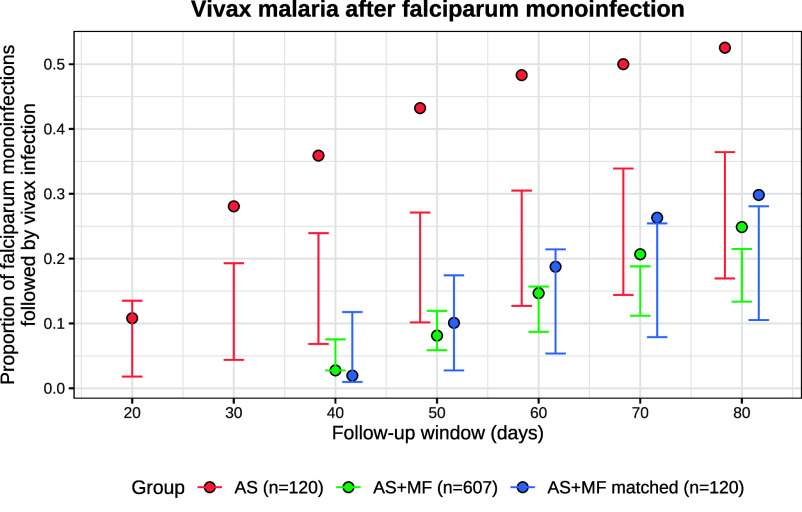
Vivax malaria following *P. falciparum* monoinfection in the SPf66 trial. Model-predicted conditional probabilities for each follow-up window are shown by the vertical bars (99% CrI). Observed proportions are shown by the filled circles. Red: artesunate (AS) monotherapy; green: artesunate-mefloquine combination therapy (AS+MF); blue: AS+MF episodes matched against AS episodes for the time of the baseline episode (to control for seasonality) and the history of *P. vivax* recurrence.

*P. falciparum* monoinfections were then stratified by schizonticidal treatment (artesunate monotherapy, n=120 vs. artesunate-mefloquine combination therapy, n=607). Artesunate is eliminated within hours of drug administration whereas mefloquine has a terminal elimination half-life of 2 to 3 wk. The rates of vivax malaria 20 to 80 d after *P. falciparum* monoinfections treated with artesunate monotherapy were substantially higher than expected under the model (red, Fig. 4). In contrast, the rates of vivax malaria in the same periods following artesunate-mefloquine were largely within the expected range (green, Fig. 4). These patterns persisted after matching treatment groups for the number of previous vivax episodes and the time of the baseline falciparum monoinfection (blue, Fig. 4).^‡^

### Overdispersion and Zero Inflation of the Hypnozoite Burden.

The model estimated considerable overdispersion in the hypnozoite burden across individuals, resulting in population heterogeneity in the risk of relapse even under the assumption of homogeneous transmission. The steady-state hypnozoite distribution is negative binomial (19) (*SI Appendix*, Appendix E.2). Assuming that our estimates for the mean (geometrically distributed) sporozoite batch size (ν=6.9) and the mean duration of hypnozoite carriage (1/η=171 d) are generalizable across endemic areas with short-latency *P. vivax*, we characterized hypnozoite burdens in individuals at steady state across a range of transmission settings. Here, we assume that the fundamental biology of the parasite has not changed in the 30 y since the trial was conducted. If *P. vivax* fundamental biology changes over time and across endemic settings, these results should be interpreted with caution.

**Fig. 5.**
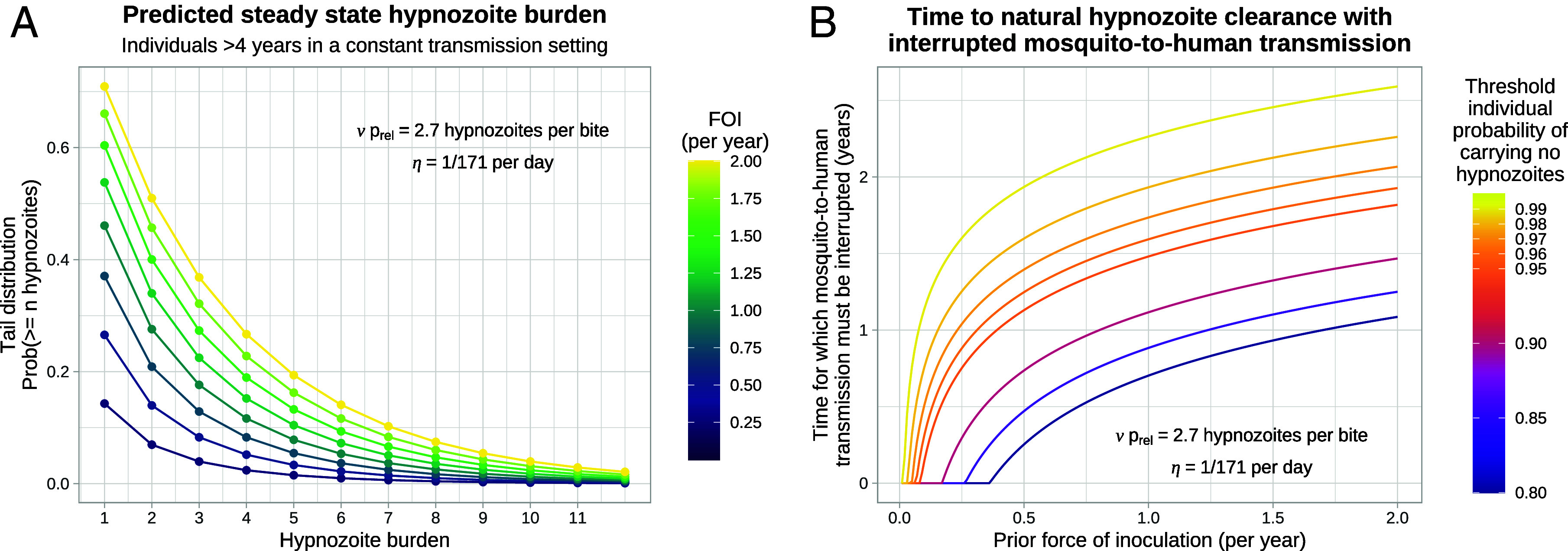
Overdispersion in the hypnozoite burdens across individuals and expected times until all hypnozoites clear naturally, as a function of the force of inoculation (FOI). Panel (*A*) shows the tail distribution for the size of the hypnozoite reservoir. Panel (*B*) shows the duration of time for which mosquito-to-human transmission must be interrupted for a individuals to clear all hypnozoites naturally with a threshold probability.

Fig. 5*A* shows the model-predicted distribution of the number of hypnozoites carried by individuals as a function of different transmission intensities (for simplicity this assumes a constant force of inoculation). The expected time until a threshold proportion of individuals no longer carry hypnozoites is determined by the overdispersion in hypnozoite carriage (Fig. 5*B*). Substantial overdispersion has important consequences for *P. vivax* elimination and control. For example, if it was possible to interrupt human-to-mosquito transmission temporarily (for example, by mass antimalarial drug treatment, or the widespread administration of ivermectin as an endectocide or deployment of an effective insecticide) and if the force of inoculation was 0.5 infectious bites per year before interruption of transmission, then individuals are predicted to carry hypnozoites with probability 27% only, but it would still take 1.6 y to eliminate hypnozoites with probability 98% in each individual (Fig. 5*B*).

### Assessing the Utility of a Serological Test for Radical Cure.

There has been recent interest in using a multiplex serological assay to identify individuals who had a recent bloodstream *P. vivax* infection (34, 35). The serological test is based on a panel of 8 *P. vivax* specific antibodies and is estimated to have >80% specificity and >80% sensitivity in detecting bloodstream infections which occurred within the previous 9 mo. Recent bloodstream infection is predictive of current hypnozoite carriage, so serological testing could allow for targeted administration of 8-aminoquinoline drugs.

**Fig. 6.**
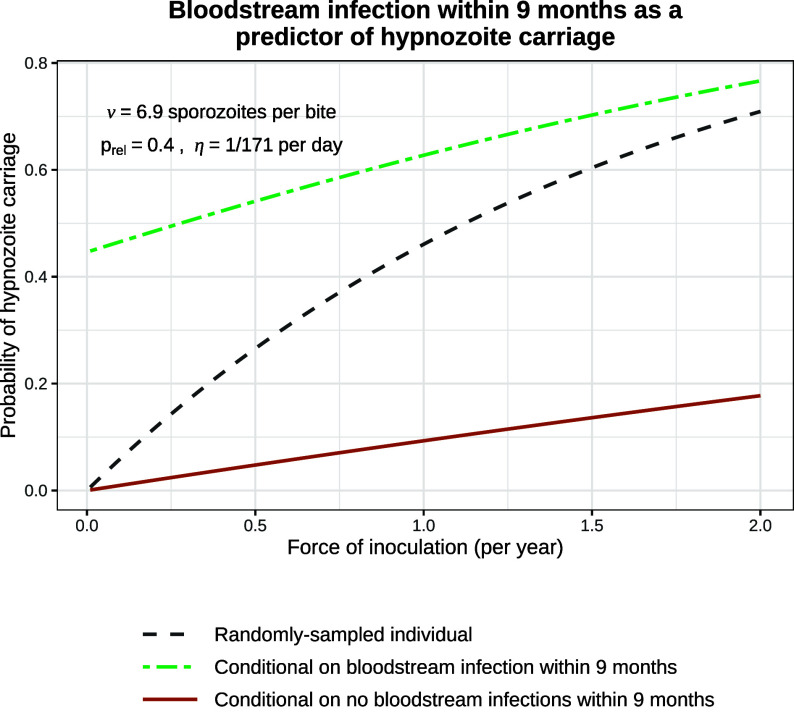
The probability of hypnozoite carriage. This compares the probability that a randomly sampled individual carries hypnozoites (dashed gray line) with the conditional probability given a bloodstream infection within the previous 9 mo (i.e. the positive predictive value, green) and the conditional probability given no bloodstream infection within the previous 9 mo (i.e. the false omission rate, maroon).

Using our calibrated model, we assessed the utility of such a test under different epidemiological scenarios. The positive predictive value of a recent bloodstream infection approaches the baseline probability of hypnozoite carriage for a randomly sampled individual as the force of inoculation increases (dashed gray vs. green line, Fig. 6). For transmission intensities lower than 0.5 infectious bites per year, individuals who have had recent bloodstream infections are more than twice as likely to harbor hypnozoites than randomly sampled individuals; for a force of inoculation of 2 bites per year, they are only 1.1 times more likely to harbor hypnozoites. The false omission rate (conditional probability of hypnozoite carriage given no recurrence occurred within the preceding 9 mo) is generally low but rises to 18% under an inoculation rate of 2 infectious bites per year (maroon line, Fig. 6). This shows the limited utility of serological testing to identify hypnozoite carriage except in near-elimination settings (36).

**Fig. 7.**
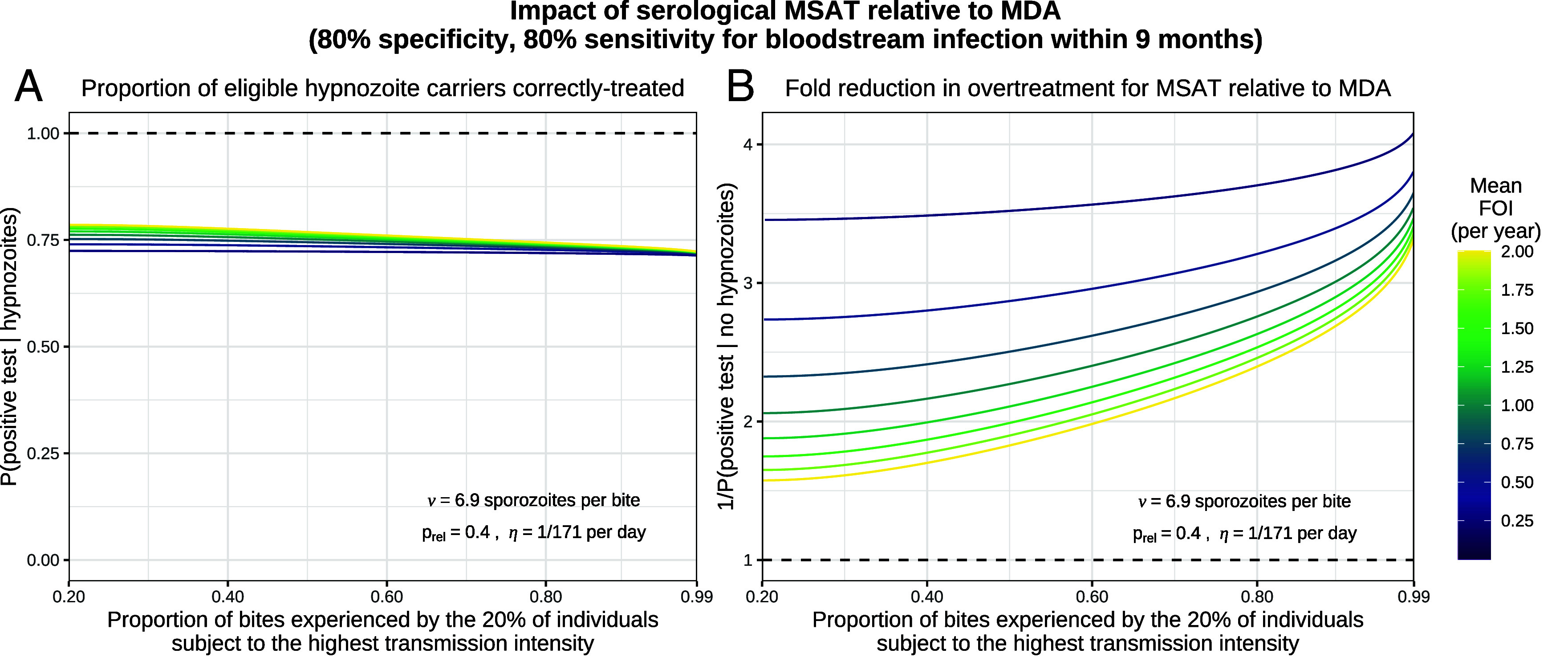
The modeled impact of serological MSAT (assuming 80% sensitivity and 80% specificity for bloodstream infection within 9 mo) relative to MDA, as a function of transmission intensity and population heterogeneity. We model each individual to be subject to a constant force of inoculation (FOI), sampled from a Gamma distribution (37) parameterized by the population mean and the proportion of bites experienced by the 20% of individuals subject to the highest transmission intensity. Panel (*A*) shows the proportion of eligible hypnozoite carriers who are correctly treated in the MSAT setting. Panel (*B*) shows the fold reduction in overtreatment for MSAT vs. MDA.

Based on these observations, we used our model to assess the theoretical impact of a serological mass screen and treat (MSAT) strategy relative to mass drug administration (MDA) (both aim to provide radical cure). As we expect much greater heterogeneity in more typical epidemiological settings, we allow the force of inoculation to be Gamma-distributed (37). Given a serological test with 80% specificity and 80% sensitivity for recent recurrence, we predict that approximately 75% of hypnozoite carriers (in the eligible population) are correctly targeted for treatment through MSAT, with this figure largely insensitive to both transmission intensity and heterogeneity (Fig. 7*A*). The reduction in overtreatment (i.e. anti-hypnozoite treatment of individuals who do not carry hypnozoites) for MSAT vs. MDA is most pronounced in low and heterogeneous transmission settings (Fig. 7*B*). For example, in a population where individuals are bitten once a year on average, but 20% of individuals receive 80% of all infective bites (Gini coefficient 0.985), we predict serological MSAT to yield a 2.8 fold reduction in overtreatment relative to MDA (in the eligible population). Given the significant risk of oxidant hemolysis in G6PD-deficient individuals with radical cure regimens, MSAT and MDA would both need to be accompanied by point-of-care G6PD testing (38). Reducing overtreatment is beneficial because it reduces both the cumulative cost of G6PD testing, and the risk of misallocation (i.e., administering radical curative treatment to a G6PD-deficient individual). It is important to stress that assessment of a complex intervention such as MSAT cannot be done only through the lens of a mathematical model. Our within-host model provides one piece in a complex cost-effectiveness framework. To characterize relative cost-effectiveness of MSAT vs. MDA in near-elimination settings (i.e. where mass treatment could plausibly be cost-effective), it is necessary to take into account the operational costs and logistic complexity (for example, serological screening without a point-of-care test requires two successive visits per individual), and the opportunity costs.

## Discussion

*P. vivax* is now the predominant human malaria parasite species in most of endemic Asia and Latin America. It is an important cause of morbidity and developmental delay in children. The main barrier to *P. vivax* elimination is relapse. Unless an effective radical cure regimen with an 8-aminoquinoline antimalarial is given, treatment of the bloodstream infection does not prevent subsequent hypnozoite activation and consequent recurrent malaria. Characterizing the within-host dynamics of relapse is important for understanding risk factors for recurrent malaria and temporal patterns of illness. It informs strategies for malaria control and elimination and methods of therapeutic assessment. We have used serial data from an intensively followed large cohort of children studied on the North-Western border of Thailand, a region of low seasonal malaria transmission. Although the average *P. vivax* inoculation rate in this setting was estimated at approximately 0.5 infectious bites per person per year, this level of transmission was sufficient to generate a high frequency of *P. vivax* infections in younger children with consequent substantial morbidity.

We showed that a simple model of relapse based on independent constant rate activation dynamics for each hypnozoite was sufficient to capture most of the inter- and intra-individual variability in recurrence patterns. Under this model, the extremely detailed SPf66 vaccine trial data allowed confident estimation that the mean duration of carriage for a single hypnozoite is approximately 6 mo. This model-dependent estimate was strongly informed by the patterns of successive recurrences observed in each individual and was robust to misspecification of the sporozoite batch size, age-stratification in the force of inoculation, and the ratio of immediately developing sporozoites to hypnozoites. Substantial unmeasured heterogeneity in transmission would bias this estimate upward, but analysis of falciparum malaria incidence suggests limited heterogeneity in this population.

A uniform force of inoculation did not explain the overall pattern of infections by age in the SPf66 trial. On the Thailand–Myanmar border, anopheline vectors largely feed outdoors around dawn and dusk (39) so behavioral differences (e.g. greater mobility as children get older, or age-related outdoor activities like play, domestic labor, and farming) may have resulted in differential exposures across age groups. Differences in body surface area or regularity of bed-net usage across age groups may have been contributing factors to a lower force of inoculation in the youngest children (22).

The exponential clock model coupled with chloroquine suppression (but not elimination) of subsequent asexual parasite growth (27) creates a cyclical pattern of recurrent malaria. Although it is intrinsically “wasteful,” in that hypnozoites may activate at a time when subsequent asexual growth is suppressed by active infection (or antimalarial treatment) and so the parasite progeny do not reach transmissible densities, the random activation model requires sporozoite inocula which are still within the range considered likely in the natural setting. The model yields testable hypotheses about *P. vivax* biology (for example the mean duration of carriage) that may be interrogated through appropriate in vivo platforms (40). Further, we provide a simple framework with which to assess the utility of public health interventions such as MSAT (34, 35).

In the SPf66 vaccine trial, both vivax and falciparum malaria were frequent in children, and mixed infections were recorded in 9% of cases. Most of the falciparum malaria cases were treated with artesunate-mefloquine which provided a long post-treatment suppression of subsequent *P. vivax* infections. However, by comparison with chloroquine, which suppresses but usually does not eliminate the relapse bloodstream infection (27), these data suggested that mefloquine does eliminate a proportion of the expected *P. vivax* relapses. This may be explained by the elimination profile of mefloquine which results in relatively high blood concentrations in the weeks following drug administration (41), whereas the chloroquine levels decline rapidly before plateauing at a relatively low level (42). This is a significant public health advantage for mefloquine which has not been appreciated previously.

In malaria endemic areas *P. falciparum* infections are commonly followed by *P. vivax* infections with an interval that is similar to the interval between a primary *P. vivax* infection and its subsequent relapse (13, 14). In this study, mefloquine appears to have eliminated many of the blood stage *P. vivax* recurrences occurring within the first month after treatment so vivax malaria recurrences after falciparum malaria were less frequent. However in the minority of symptomatic falciparum malaria episodes (n=137) treated with artesunate monotherapy (which provides limited post-treatment prophylaxis), we observed higher than expected recurrence rates, consistent with previous studies indicating significantly different rates following slowly vs. rapidly eliminated antimalarial treatment (14). In particular, the rate of early *P. vivax* recurrence following artesunate monotherapy was much higher than could be explained by random hypnozoite activation. While these results are partially confounded by seasonality and do not provide definitive proof that hypnozoites can be activated from external triggering events such as febrile illness, this observation is not explained by the exponential clock model and is compatible with external triggering. Determining causality is not possible from these data. Other factors could be responsible for activation, such as artesunate itself. However, high rates of relapsing vivax malaria have also been observed after falciparum infections treated with quinine (13), which has a different mode of action. This suggests that the mechanism of hypnozoite activation is independent of the primary antimalarial drug treatment. Overall, our model provides a general framework for characterizing the importance of external triggering, with potential implications for treatment: for example, radical curative treatment in patients with falciparum malaria reduces the risk of vivax recurrence (13, 43).

There are several factors which may have influenced the observed vivax malaria patterns in the SPf66 trial, and thus affect the interpretation of some inferred model parameters. The daily assessment means that the duration of illness was nearly always less than 24 h, which is unusually brief for malaria and shorter than in clinical trials of antimalarial treatment. This artificial situation, necessary for the vaccine trial, would have substantially attenuated any disease modification in hypnozoite activation risk. High quality microscopy ensured that symptomatic malaria would have almost certainly been identified, but presumably also captured incidental parasitemias with other febrile illnesses. This is particularly plausible for *P. vivax*, given primaquine was not given to prevent relapse. In therapeutic studies approximately half the relapses of *P. vivax* are not accompanied by fever, and recent studies in areas of similar transmission intensity in this region indicate high rates of asymptomatic parasitemia, even in young children, some of which are detectable by good microscopy (44). A key correlate of acute febrile malaria is thrombocytopenia (platelet count <150,000$/\;{\text{mm}}^{3}$) (45); however, across clinical consultations in the SPf66 cohort, thrombocytopenia was only present in 34% with a diagnosis of vivax monoinfection, 41% with a diagnosis of falciparum monoinfection, and 42% with a diagnosis of mixed infection (*SI Appendix*, Appendix A.3). Although the population was aggregated together, there were no physical barriers. As such, movement, individual behaviors, geographic location, and use of malaria preventive measures may have given rise to population heterogeneity in inoculation rates. However, the plausible degree of transmission heterogeneity in the SPf66 vaccine trial (with an upper bound based on the incidence of symptomatic falciparum malaria, *SI Appendix*, Appendix A.5) is unlikely to yield a substantial departure from our estimates (*SI Appendix*, Appendix D.2).

In conclusion, a simple model of *P. vivax* relapse assuming constant rate activation of liver hypnozoites was sufficient to explain the majority of variation in relapse patterns in a low transmission setting co-endemic for falciparum and vivax malaria. This does not exclude other processes such as disease activation, which would explain satisfactorily the elevated rate of *P. vivax* recurrence following symptomatic *P. falciparum* monoinfection, but for the most part does not require them. This model can be used to help assess the utility of public health interventions for the control and elimination of vivax malaria, and provides a methodology for the analysis of detailed prospective malariometric studies.

## Materials and Methods

### Data from the SPf66 Vaccine Trial.

The peptide polymer SPf66 was among the earliest synthetic vaccine candidates for falciparum malaria (46). Early field studies gave rise to inconsistent efficacy estimates (47–49). Putative evidence of the ineffectiveness of the SPf66 vaccine against symptomatic malaria was provided by a randomized double-blind trial conducted between October 1993 and July 1995 on the Thailand–Myanmar border, a region co-endemic for *P. falciparum* and *P. vivax* (17). In retrospect, this vaccine trial constitutes the most detailed cohort study of vivax malaria ever conducted.

#### Study design.

Over a study period spanning 21 mo, daily home or school visits were conducted for 1,344 children aged 2 to 15 y of age living in Shoklo camp—a community of refugees of Karen ethnic minority. Children exhibiting signs of illness were referred to outpatient clinics, where malaria was subsequently diagnosed using light microscopy. Absences from the camp were duly documented. Additional passive surveillance data were also collected, in thorough cross-sectional surveys conducted at two to three month intervals. We did not use data from the cross-sectional surveys in this analysis as the interpretation of an asymptomatic microscopic infection was limited by the proximity of the pyrogenic threshold for vivax infection [∼180/μL (39)] to the limit of detection of light microscopy. We focused instead on the sequence of symptomatic malaria episodes recorded for each child, assuming perfect detection of symptomatic episodes during periods of active clinical follow-up.

#### Antimalarial treatment.

Confirmed symptomatic cases of malaria were promptly treated with supervision; asymptomatic episodes detected through cross-sectional surveys were not treated. Typically, vivax monoinfections were treated with chloroquine (25 mg base/kg over 3 d), while uncomplicated falciparum monoinfections or mixed infections were treated with artesunate-mefloquine combination therapy (artesunate 4 mg/kg per day for 3 d and a single dose of mefloquine 25 mg base/kg on day 2 of treatment), with some exceptions; most notably, the administration of artesunate monotherapy to some children with hyperparasitemic falciparum malaria (see *SI Appendix*, Appendix A.1 for details). Neither chloroquine nor mefloquine appear to have conferred any post-treatment protection against falciparum malaria, consistent with contemporaneous evidence of widespread multidrug-resistant *P. falciparum* (26). Differential patterns of extended prophylactic protection against *P. vivax* were apparent following the administration of the slowly eliminated antimalarials chloroquine and mefloquine: While chloroquine appeared to have given rise to a “prophylactic bunching” phenomenon, whereby residual drug levels (below the minimum inhibitory concentration) lead to the delayed manifestation rather than suppression of bloodstream infection (27), mefloquine appeared to have eliminated *P. vivax* bloodstream infections for an extended duration of time (*SI Appendix*, Fig. A.1 and Appendix A.1). We adjusted for post-treatment prophylaxis by defining a fixed period of prophylactic protection for each drug regimen, during which all emerging bloodstream infections are eliminated. This was assumed to span 15 d, with two exceptions: Chloroquine monotherapy was assumed to provide no protection against *P. falciparum*, while mefloquine was assumed to eliminate all emerging *P. vivax* bloodstream infections for an extended period of 35 d. Any consultation that overlapped with the prophylactic protection period of a previous episode was flagged as a possible treatment failure (see *SI Appendix*, Appendix A.1.4 for details).

#### Incidence of symptomatic malaria.

We computed the incidence of symptomatic malaria after removing likely treatment failures and adjusting the person-time at risk for periods of prophylactic protection (in addition to left/right-censoring and documented absences from the camp). To assess age structure, we calculated age-stratified incidence rates (i.e. the total number of symptomatic episodes divided by the cumulative person-years at risk for each age group) with bootstrap resampling. We applied the Kruskal–Wallis test and the non-parametric Mack-Wolfe test for umbrella alternatives (with unknown peak) (50) to the incidence per individual (i.e. the number of symptomatic episodes divided by the time at risk) within each age group (*SI Appendix*, Appendix A.4). To estimate a plausible upper bound for transmission heterogeneity in the SPf66 vaccine trial, we calibrated a negative binomial (Gamma-Poisson mixture) model to the incidence of symptomatic falciparum malaria (*SI Appendix*, Appendix A.5).

### A Theoretical Within-Host Framework for Hypnozoite Accrual.

We embedded the exponential clock model of White et al. (16) in a stochastic framework of sporozoite inoculation from repeated mosquito bites. This model was formulated as an open network of infinite server queues, generalizing the construction in ref. 19 to capture sporozoite “destiny” (immediate development vs. hypnozoite formation). The original model in ref. 16 accommodated a constant death rate for each hypnozoite to capture hepatocyte turnover. Since hypnozoite death is unidentifiable on the basis of temporal recurrence data, we formulated a reparameterized system restricted to successful sporozoites—that is, sporozoites that either undergo immediate development or form hypnozoites that eventually activate to give rise relapses (see *SI Appendix*, Appendix B.1 for details of this reparameterization, and an explicit link to ref. 19).

#### Model structure.

**Fig. 8.**
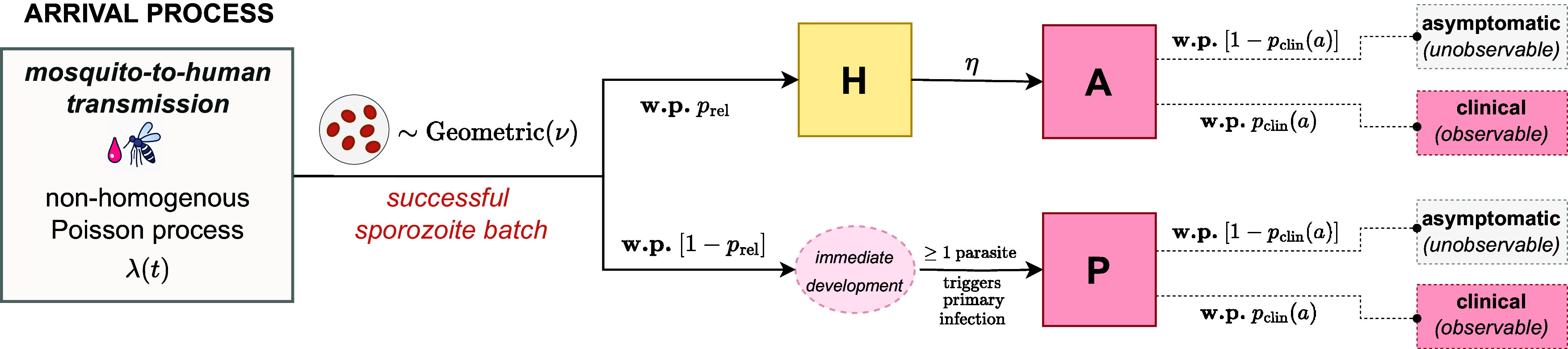
Schematic of the open network of infinite server queues used to model the burden of bloodstream infection (see *SI Appendix*, Appendix B.1 for a link to ref. 19). Adapted from figure 1 of ref. 19. **w.p.**: with probability.

The construction of the within-host framework, illustrated in Fig. 8, is as follows:

- The timing of infective bites is modeled with a non-homogeneous Poisson process characterized by the time-dependent rate λ(t).
- The number of successful sporozoites S established by each infective bite is modeled to follow a geometric distribution, with mean ν and state space $Z_{\geq 0}$.
- With probability $p_{\;\text{rel}}$, each (successful) sporozoite forms a hypnozoite that is destined to activate (state H); otherwise, it undergoes immediate development.
- The immediate development of one or more sporozoites within a batch gives rise to a primary infection (state P).
- The time to activation for each hypnozoite—or equivalently, the duration of hypnozoite carriage—is modeled to be exponentially distributed with mean 1/η (with hypnozoite activation represented by a transition from state H to A).

Treating age as a proxy for previous malaria exposure, anti-disease immunity is modeled as an age-dependent masking mechanism. For an individual of age a at enrollment, each immediately developing sporozoite batch (i.e. arrival into node P) and hypnozoite activation event (i.e. arrival into node A) in the study period is assumed to give rise to clinical symptoms with some probability $p_{\;\text{clin}}\left(a\right)$; the time to manifestation of clinical symptoms is not modeled. We assume the functional form

[1] $$\begin{array}{c} p_{\;\text{clin}}\left(a\right)=1-\left(1-\rho \right)(\frac{a-a_{\;\text{min}}}{a_{\;\text{max}}-a_{\;\text{min}}}{)}^{\gamma }, \end{array}$$

whereby

- $p_{\;\text{clin}}\left(a_{\;\text{min}}\right)=1$ for the youngest age group in the study ($a_{\;\text{min}}=2$ y olds);
- the parameter ρ corresponds to the proportional reduction in the probability that a bloodstream infection will be symptomatic across the age range of the study (i.e. between $a_{\;\text{min}}=2$ and $a_{\;\text{max}}=15$ y of age); and
- the exponent γ modulates the steepness/shape of the (monotonically decreasing) curve, which is concave up in the case γ<1, linear in the case γ=1 and concave down if γ>1.

This construction does not account for heterogeneity in anti-disease immunity within age groups, arising from heterogeneity in prior exposure. The proportion of symptomatic infections in the 2-y-old group is not identifiable^§^ but we assume it is 1. We therefore interpret $p_{\;\text{clin}}$ as the probability that a bloodstream infection is symptomatic *relative* to the average 2 y old (the reference group). If a large proportion of cases in 2 y olds were in fact asymptomatic, we would underestimate the sporozoite batch size.

Metrics of epidemiological interest were derived analytically under this stochastic within-host framework (*SI Appendix*, Appendix E), under the simplifying assumption that the hypnozoite reservoir had reached stationarity under a constant force of inoculation λ (prior to the study period; this has very little impact on the parameter inference). Accommodating population heterogeneity by randomly sampling the force of inoculation for each individual from a Gamma distribution (37) is also analytically tractable.

### An Inferential Framework Tailored to the SPf66 Vaccine Trial.

We constructed an inferential framework, tailored to the SPf66 dataset, that allowed us to compute the exponential clock model likelihood for multiple observed recurrent vivax episodes. For this to be tractable we discretized the study period into $n_{\;\text{obs}}=65$ windows, each of length T=10 d. The force of inoculation for *P. vivax* was assumed to be piecewise constant over each window, enabling the derivation of an analytic likelihood and substantially simplifying inference. Age is the key covariate in the model, serving as a determinant of both the baseline hypnozoite burden (since it determines the time over which the reservoir accrues before the start of the study) and the degree of anti-disease masking.

#### Context-dependent observational model.

##### Discretized vivax infection states adjusted for post-treatment prophylaxis.

In addition to anti-disease immunity (a structural feature of the within-host model), hypnozoite activation and immediate sporozoite development events may have been masked (i.e. unobserved) due to censoring or post-treatment prophylaxis. For each child, we constructed a binary sequence of clinical infection states, indicating whether or not a symptomatic vivax episode was recorded for each 10 d window. We masked any windows in which a child was actively followed up for 5 d or less (due to either left- or right-censoring, or a documented absence from the camp). We additionally modeled post-treatment prophylaxis as follows (with some exceptions in the vicinity of possible treatment failures; see *SI Appendix*, Appendix A.6.1). For every window i for which antimalarial treatment was administered, we masked window (i+1); if mefloquine was administered, we additionally masked windows (i+2) and (i+3) to allow for an extended period of prophylactic protection (i.e. we assumed that merozoites released from the liver during this period were unable to successfully establish bloodstream infections because drug levels were high enough to eliminate asexual parasites resulting from hepatic schizogony [10,000 to 100,000 parasites]). If chloroquine was administered in window i and a symptomatic vivax episode was recorded in window (i+3), then we grouped together windows (i+2) and (i+3) [i.e. we allowed for the potentially delayed manifestation of clinical symptoms following any hypnozoite activation or immediate sporozoite development events during this period, due to an asexual parasite multiplication rate that was temporarily dampened by residual drug levels but still exceeded one (27)]. Discretized vivax infection states for children aged 7 at enrollment are visualized in *SI Appendix*, Fig. A.6.

##### Seasonality in the force of inoculation.

We used the incidence of symptomatic falciparum malaria as a direct proxy of the *P. vivax* mosquito inoculation rate. To infer relative seasonal fluctuations in the force of inoculation for *P. vivax* over the study period, we fitted a smoothing spline to the incidence of symptomatic falciparum malaria calculated in 20 d windows, with a moving average across half-windows (see *SI Appendix*, Appendix A.6.2 for details); in the main model fit we then estimated a scaling factor governing the ratio of vivax to falciparum inoculation rates (this was done as a two-stage process without uncertainty propagation). This scaling factor was estimated separately before and after April 1994 (day 200 of the study period), to account for variation in the relative burden of vivax vs. falciparum malaria following the camp-wide shift in treatment regimen for falciparum malaria (24, 25). We assumed a constant force of inoculation prior to the study period, i.e., seasonality and systematic changes in vivax transmission in the years preceding the study were not modeled. We did not account for heterogeneity or age-stratification in the force of inoculation.

#### Analytic model likelihood.

Under the theoretical framework, for an individual of age a at enrollment, we modeled hypnozoite accrual for duration a, before considering the number of hypnozoite activation and immediate development events $N_{i}\left(a\right)$ in each window $i=1,\cdots ,n_{\;\text{obs}}$ of the study period. We characterized the joint distribution of N(a) from first principles, by deriving a multivariate probability generating function (PGF) (*SI Appendix*, Appendix B.2). Our argument mimics the approach of refs. 19 and 51, 52, 53 and others: We condition first on the time course of each sporozoite, followed by the size of an incoming sporozoite batch and finally, the sequence of bite times. For an individual of age a, the likelihood of clinical symptoms in a set of windows I (which may be aggregated due to prophylactic bunching) takes the form

$$\begin{array}{c} P\left(\;\text{clinical symptoms in}\;I\;|\;N\left(a\right)\right)=1-{\left(1-p_{\;\text{clin}}\left(a\right)\right)}^{\sum_{i\in I}N_{i}\left(a\right)}. \end{array}$$

Application of the inclusion–exclusion principle to the multivariate PGF of N(a) allows the likelihood of a given sequence of symptomatic episodes to be recovered analytically (*SI Appendix*, Appendix B.2). The time complexity of evaluating the likelihood scales exponentially with the number of symptomatic episodes per child; computational constraints, coupled with numerical instability arising from catastrophic cancellation, yield a practical upper bound of 13 symptomatic episodes per child for our implementation, which is the maximum observed in the SPf66 trial.

#### Bayesian inference using the Metropolis–Hastings algorithm.

We performed Bayesian inference using the Metropolis–Hastings algorithm. The proportion of hypnozoites (vs. immediately developing forms) was set to $p_{\;\text{rel}}=0.4$, informed by recent in vivo experiments on the Chesson strain of *P. vivax* (28). We thus generated estimates for 6 parameters: the hypnozoite activation rate η; the mean sporozoite batch size ν; the average force of inoculation $\Lambda_{1}$ and $\Lambda_{2}$ before and after day 200 of the study period respectively; and the parameters ρ and γ governing the age-dependent anti-disease masking probability (Eq. **1**). We used flat improper priors on the non-negative real line for the parameters $\left\{\Lambda_{1},\Lambda_{2},\nu ,\eta \right\}$ but informative normal priors for γ and ρ:

$$\begin{array}{c} \log\left(\gamma \right)∼N\left(0,0.6^{2}\right)\;\;\text{logit}\left(\rho \right)∼N\left(0,0.7^{2}\right). \end{array}$$

The prior for the probability of symptomatic infection ρ at age 15 relative to age 2 had median value 0.5, with 95% mass in the interval [0.2, 0.8]. The prior for the shape parameter log(γ) of the anti-disease masking curve was symmetric around zero (i.e. linear decline), with 95% mass in the interval [−1.18, 1.18]; the transformation log(γ)↦−log(γ) reflects the curve along the line of identity. We implemented a symmetric (rectified) normal proposal distribution. Details are provided in *SI Appendix*, Appendix C.1.

#### Posterior predictive checks.

To perform posterior predictive checking, we simulated symptomatic vivax episodes under 2,000 parameter combinations sampled from the posterior uniformly at random (without replacement). Simulated cohorts had the same age structure as the SPf66 trial. The sequence of hypnozoite activation and immediate sporozoite development events under the exponential clock model was obtained through direct stochastic simulation (*SI Appendix*, Appendix C.2.1). We adjusted for anti-disease masking to recover a discretized sequence of binary clinical infection states over T=10 d windows. We then imposed an observational model, accounting for lapses in clinical follow-up (informed by patterns of left- and right-censoring, and documented camp absences in the SPf66 cohort) and post-treatment prophylaxis (with identical parameters to chloroquine). *P. falciparum* episodes were not simulated. We compared age structure and seasonal fluctuations in the incidence of symptomatic vivax malaria for these simulated cohorts against observed data.

#### Vivax after falciparum monoinfection.

To interrogate whether the exponential clock model could explain observed rates of *P. vivax* recurrence following falciparum monoinfection, we derived the probability of observing a symptomatic vivax recurrence in 20 to 80 d windows following each falciparum monoinfection (due to either spontaneous hypnozoite activation and/or immediate sporozoite development). In doing so, we conditioned on the recorded history of vivax recurrence prior to the baseline falciparum monoinfection. We additionally accounted for censoring due to lapses in clinical follow-up and post-treatment prophylaxis following the treatment of falciparum malaria in each relevant follow-up window. Given the resultant set of probabilities, we modeled the number of falciparum monoinfections followed by a vivax recurrence within the window using a Poisson binomial distribution (i.e. as a sum of independent, but non-identically distributed Bernoulli random variables). We computed 99% credible intervals for the proportion of falciparum monoinfections followed by *P. vivax* recurrence based on 2,000 parameter combinations sampled uniformly at random from the posterior. Details are provided in *SI Appendix*, Appendix C.3.

We stratified baseline episodes based on treatment with artesunate monotherapy vs. artesunate-mefloquine combination therapy, given empirical evidence that early emerging bloodstream infections were suppressed following the administration of mefloquine (see Kaplan–Meier curves for time to vivax recurrence following falciparum monoinfection, *SI Appendix*, Fig. A.2). We removed falciparum monoinfections in the vicinity of treatment failure (*SI Appendix*, Appendix A.1.4) and considered baseline episodes up until April 1995 (day 570 of the study, to yield adequate clinical follow-up) leaving n=120 falciparum monoinfections treated with artesunate monotherapy and n=607 treated with artesunate-mefloquine combination therapy. For doubly robust analysis, we additionally matched the treatment groups by the number of previous vivax episodes and the time of the baseline falciparum monoinfection (to control for seasonality) using the nearest neighbor method implemented in the R function MatchIt::match.data (54).

#### Sensitivity to model misspecification.

To assess the sensitivity of our parameter estimates to model misspecification, we conducted several supplementary analyses. Sporozoite inocula may be over- or under-dispersed relative to the geometric distribution. We thus performed a series of simulation studies, whereby data were simulated with negative binomial sporozoite batches, but inference was performed under the assumption of geometric sporozoite batches (*SI Appendix*, Appendix D.1). To evaluate the consequences of unmeasured population heterogeneity, we performed an additional simulation study whereby data were simulated under a heterogeneous Gamma-distributed force of inoculation (37); but inference was performed under a misspecified model predicated on a homogeneous force of inoculation, for a range of parameter values that may be plausible in low transmission settings (*SI Appendix*, Appendix D.2). Additionally, the ratio of hypnozoites to immediately developing forms for the Chesson strain of *P. vivax* (28) may not be representative of South East Asian strains. As such, we conducted a similar sensitivity analysis for the hypnozoite fating probability $p_{\;\text{rel}}$ (*SI Appendix*, Appendix D.3). In the absence of clear age structure in the incidence of symptomatic falciparum infection (*SI Appendix*, Fig. A.4) it is reasonable to assume there was no age stratification in the mosquito inoculation rate for 2 to 15 y olds; however, infants under the age of 2 may have been subject to lower exposure (31, 32). To accommodate potential age-stratification in mosquito inoculation exposures for infants <2 y vs. children >2 y, we recalibrated the model with the force of inoculation for infants scaled down by a series of fixed factors (*SI Appendix*, Appendix D.4).

## Supplementary Material

Appendix 01 (PDF)

## Data Availability

All data and code are openly available on a GitHub repository: https://github.com/somyamehra/SPf66 (55).

## Footnotes

^*^Unless transmission heterogeneity varied with age, systematic differences across age groups would not be expected under this time scale of hypnozoite carriage even in the presence of population heterogeneity.

^†^In individuals not enrolled in the SPf66 trial, uncomplicated falciparum malaria in the camp was largely treated with mefloquine monotherapy until early 1994, with associated high rates of treatment failure (25, 33): This biases our estimates of seasonality in mosquito inoculation, and seasonal fluctuations in the incidence of symptomatic vivax malaria are poorly captured, particularly before April 1994 (*SI Appendix*, Fig. C.3 and Appendix C.2.2).

^‡^Since artesunate monotherapy was usually given to hyperparasitemic children, there may be systematic variation in prior exposure and seasonal confounding between these groups. Matching can correct for this confounding.

^§^Focusing on the subset of hypnozoites that cause symptomatic relapse, an individual of age a accrues batches of mean size $\nu p_{\;\text{rel}}p_{\;\text{clin}}\left(a\right)$ over duration a. As such, the proportion of symptomatic infections in the baseline age group is not structurally identifiable on the basis of data for symptomatic relapse alone. Given the predominance of relapse, practical identifiability issues would plausibly persist despite the additional contribution of primary infections.
